# Trends in the prevalence and incidence of ankylosing spondylitis in South Korea, 2010–2015 and estimated differences according to income status

**DOI:** 10.1038/s41598-018-25933-4

**Published:** 2018-05-16

**Authors:** Jin-Sung Park, Jae-Young Hong, Ye-Soo Park, Kyungdo Han, Seung-Woo Suh

**Affiliations:** 1 Department of Orthopedics, Korea University Ansan Hospital, Ansan-si, South Korea; 2 Department of Orthopedics, Hanyang University Guri Hospital, Guri-si, South Korea; 3 Department of Biostatistics, Biomedicine & Health Sciences, Catholic University, Seoul, South Korea; 4 Scoliosis Research Institute, Department of Orthopedics, Korea University Guro Hospital, Seoul, South Korea

## Abstract

The aim of this study is to determine the prevalence and incidence of ankylosing spondylitis (AS) in South Korea, 2010–2015. This study was conducted using the Health Insurance Review Agency (HIRA) database, which includes information on every patient diagnosed with AS. The incidence and prevalence of AS were evaluated by age, sex, and income status. The prevalence increased linearly by 7.7% annually, i.e., 31.62 in 2010 to 52.30 in 2015 (per 100,000 persons). During the study period, the incidence was 6.34 per 100,000 person-years. The prevalence peaked for both men and women in the age range 30–39 years. Incidence peaked for men in the age range 20–29 years, but peaked for women between ages 70 and 89. AS was 3.6 times more prevalent in men than in women, and the incidence in men was 2.1 times greater than in women. With respect to income status, the prevalence and incidence of AS were 3 times greater and 5 times greater, respectively, in medical aid recipients compared to individuals with other income levels. The trend of increasing AS prevalence and the observation that 14.3% of all patients newly diagnosed with AS are medical aid recipients have significant implications for healthcare planning.

## Introduction

Ankylosing spondylitis (AS) is a chronic inflammatory disease that can potentially lead to disabilities and skeletal disorder^1^. Disease onset is typically in third decade of life, however, it is reported that the diagnosis is on average about 8 to 11 years delayed compared to when first symptoms occur^2,3^. Physical activity is often limited for patients with AS, and their quality of life, including work performance, deteriorates as the disease progresses^4,5^. This extended period during which the patients has AS places a burden on both the patient and on the national health economy as a whole^6^. Accurate prevalence estimates that consider socioeconomic aspects are important for optimal healthcare planning. However, in contrast to rheumatoid arthritis, relatively few studies of the prevalence of AS have been performed.

There have been six hospital-based studies that examined the prevalence of AS using medical records with large sample sizes (population sample of more than 100,000) until now^7–12^. Five of these studies took place in European countries; in these studies, the reported prevalence ranged between 29.5 and 262.6 per 100,000 persons^7–11^. The other study was conducted in Asia; however, its diagnosis criteria included the old Rome criteria, which differ from the more widely employed modified New York criteria; this study reported the lowest prevalence (6.5 per 100,000 persons)^12^. It is believed that patients with AS stand at lower socioeconomic status because patients with AS exhibit poor functional outcome in terms of work activity^13,14^. However, to the best of our knowledge, no epidemiologic studies have assessed the prevalence and incidence according to income status.

The aims of this study were: to use a nationwide database (1) to evaluate the prevalence and incidence of AS in South Korea, including temporal trends there of; and (2) to determine whether the prevalence and incidence of AS differ according to income status.

## Methods

### Data source

This study used a nationwide data set available in the Health Insurance and Review Agency (HIRA) that was collected from 2010 to 2015. In South Korea, the government has implemented an obligatory National Health Insurance (NHI) system, and 97% of the population is covered by the NHI system, and patients pay only about 30% of the total medical cost for health care services. Health care institutions submit claims data for health care services, including diagnosis and comorbidity codes classified by the International Classification of Diseases, 10^th^ revision (ICD-10); demographic information; admission and ambulatory care; prescription records; procedure codes; and direct medical costs to the HIRA. These claims are used to request reimbursement for the remaining 70% of the total medical cost from the NHI. The remaining 3% of the population with very low income are covered by the Medical Aid Program and are also reviewed by the HIRA. Therefore, the medical records of almost all patients at health care institutions are prospectively integrated into the HIRA database.

Moreover, the NHI has included AS in the Rare Intractable Diseases (RIDs) registration program since 2009. All patients with an RID are required to have their diagnosis certified by a physician through the uniform criteria distributed by the NHI. The NHI reviews the RID program application again to ensure the diagnostic criteria are met. Patients with an RID receive more coverage and pay only about 10% of the total medical cost. Their claims for health care services are also recorded in the HIRA database. This process ensures that diagnoses for the RID program are reliable.

### Data collection

We filtered the HIRA data to identify all patients with AS who were entered in the database from January 2010 to December 2015. The ICD-10 codes for AS in the RID program (M45.0–45.9) were used. In the RID program, patients were diagnosed with AS if they met the modified New York criteria^15^. These clinical criteria include:(1) low back pain and stiffness for at least 3 months that was improved by exercise and not relieved by rest; (2) limitation motion of the lumbar spine in both the sagittal and frontal planes; and (3) limitation of chest expansion relative to values normal for age and sex. The radiological criterion is grade ≥II bilateral sacroiliitis or grade III to IV unilateral sacroiliitis. Definite AS is diagnosed if the radiological criterion plus 2 of the 3 clinical criteria are present. Data from the total South Korean population from 2010 to 2015 was obtained from the Korea National Statistical Office. The total population per year includes the number of births, deaths, and net immigrants from the previous year. The overall population ranged from 50.17 to 51.57 million during the study period.

### Ethical statement

This study was approved by the Institutional Review Board of the HIRA Research Ethics Committee of South Korea. An informed-consent exemption was granted by the board.

### Statistical analysis

Data analyses were conducted with SAS version 9.3 (SAS Institute, Cary, NC, USA). The annual prevalence was calculated as the number of patients with AS divided by the total population for a given year. The annual incidence was calculated as the number of patients with newly diagnosed AS for a given year divided by the total population of at-risk individuals in that year who did not have a history of visiting a health care institution for AS since January 2002. The annual prevalence and incidence of AS during 2010–2015 were calculated and evaluated according to age, sex, and income status. All people covered by the NHI system pay a premium depending on their income levels (20 levels total) and the rest that are insured by the Medical Aid Program do not have to pay any premiums. Using this information, we were able to calculate the prevalence and incidence based on income status. Prevalence was presented as per 100,000 persons and incidence was presented as per 100,000 person-years in the total population with 95% confidence interval (CI). Trends over time were analyzed using Poisson regression. Direct standardization by age and sex was computed using the population estimate for 2010.

## Results

### Trends in prevalence and incidence

**Figure 1:**
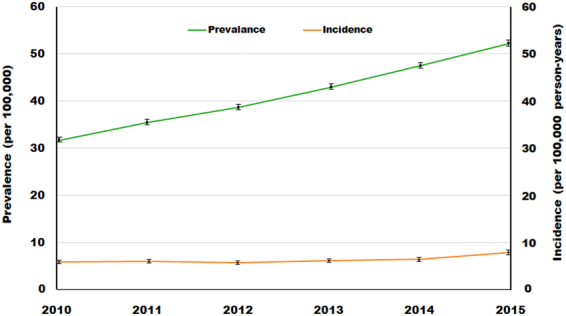
Annual prevalence and incidence from 2010 to 2015.

The prevalence and incidence trends are shown by year in Table 1 and Fig. 1. The prevalence increased linearly by 7.7% annually, i.e., 31.62 (95% CI, 31.13 to 32.11) in 2010 to 52.30(95% CI, 51.68 to 52.92) in 2015 (per 100,000 persons). However, the incidence remained similar from 2010 to 2012 (5.74 to5.65, respectively, per 100,000 person-years). Thereafter, the incidence increased slightly from 5.65 (95% CI, 5.45 to 5.86) in 2012 to 7.85 (95% CI, 7.61 to 8.09) in 2015 (per 100,000 person-years). The incidence during the study period was 6.34(95% CI, 6.27 to 6.42) per 100,000 person-years.

**Table 1:** Annual trends in prevalence rate (PR) and incidence rate (IR) from 2010 to 2015.

**Year**	**Prevalence**				**Incidence**			
	Total population	Number of prevalent cases	Crude PR, per 100,000 (95% CI)	Standardised PR^a^, per 100,000 (95% CI)	Adjusted Total population^b^	Number of incident cases	Crude IR, per 100,000 (95% CI)	Standardised IR^a^ per 100,000 (95% CI)
2010	50166793	16247	32.39 (31.89–32.88)	31.62 (31.13–32.11)	50130173	2904	5.79 (5.58–6.00)	5.74 (5.53–5.94)
2011	50445164	18257	36.19 (35.67–36.72)	35.48 (34.97–36.00)	50406084	3012	5.98 (5.76–6.19)	5.92 (5.71–6.13)
2012	50763154	20034	39.47 (38.92–40.01)	38.78 (38.24–39.31)	50721489	2894	5.71 (5.5.0–5.91)	5.65 (5.45–5.86)
2013	51013675	22242	43.60 (43.03–44.17)	42.97 (42.40–43.53)	50969639	3133	6.15 (5.93–6.36)	6.12 (5.91–6.33)
2014	51281917	24724	48.21 (47.61–48.81)	47.48 (46.89–48.07)	51235260	3313	6.47 (6.25–6.69)	6.43 (6.21–6.65)
2015	51574044	27419	53.16 (52.54–53.79)	52.30 (51.68–52.92)	51524226	4089	7.94 (7.69–8.18)	7.85 (7.61–8.09)

^a^Standardized by age and sex for the 2010 population.

^b^Individuals who did not have ankylosing spondylitis at the start of the given year.

### Prevalence and incidence according to age and sex in 2015

**Figure 2:**
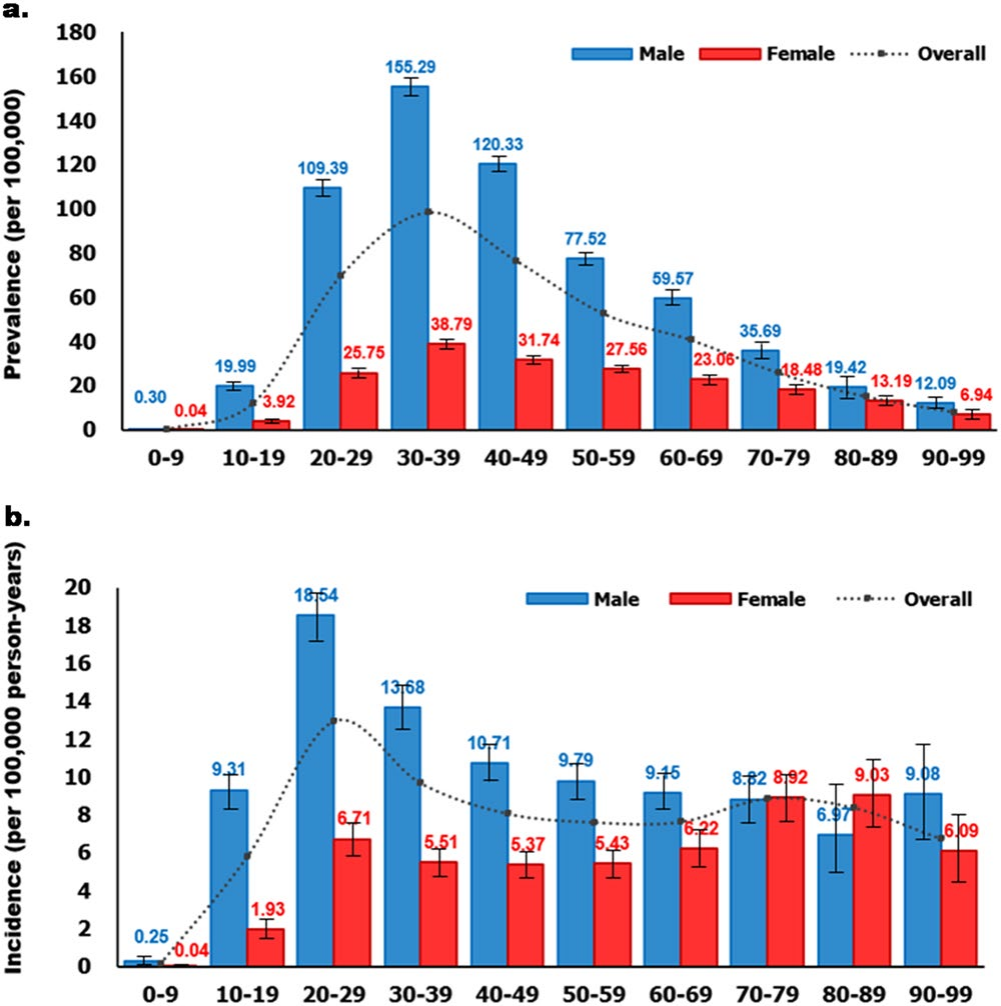
Age- and sex-specific (**a**) prevalence and (**b**) incidence in 2015.

The prevalence of AS in men (83.11 per 100,000 persons) was 3.6 times that in women (23.16 per 100,000 persons). The prevalence peaked for both men and women in individuals aged 30–39 years. Thereafter, the prevalence decreased with age. Men showed a higher prevalence than women in all age groups (Fig. 2a).

The incidence in men (10.71 per 100,000 person-years) was 2.1 times that in women (5.16 per 100,000 person-years). For men, the incidence peaked in individuals aged 20–29 years. Thereafter, the incidence decreased with age. In women, the incidence had a bimodal age distribution, peaking in two different groups (20–29 years and 70–89 years). The incidence in women aged 70–89 years was higher than that in women aged 20–29 years (Fig. 2b).

**Figure 3:**
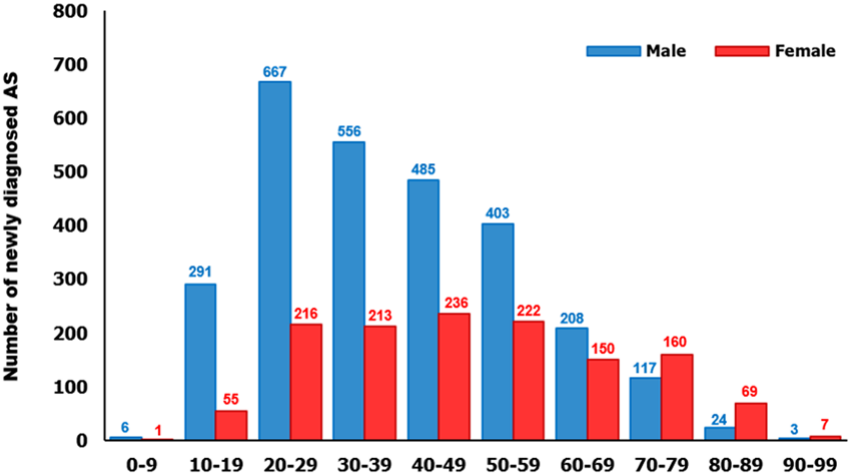
Age- and sex-specific patients newly diagnosed with AS in 2015.

Diagnosis also peaked in men aged 20–29 years, while diagnosis for women peaked in individuals aged 40–49 years. Of note, diagnosis of women in their 40 s and later was very common, indicating that diagnosis was more common at a much older age for women than men (Fig. 3).

### Prevalence and incidence according to income status in 2015

**Figure 4:**
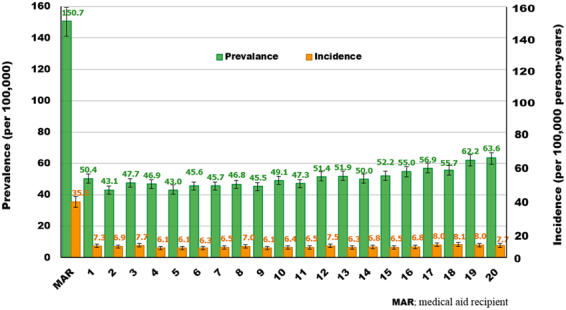
Prevalence and incidence in 2015 according to income status.

The highest prevalence and incidence were found in medical aid recipients. The prevalence in medical aid recipients was about 3 times that in individuals with all other income statuses. The incidence in medical aid recipients was about 5 times that in individuals with all other income statuses. All income statuses, with the exception of medical aid recipients, showed similar prevalence and incidence. About 14.3% (584/4089) of all newly diagnosed patients with AS were medical aid recipients (Fig. 4).

## Discussion

This is the first nationwide epidemiologic study of AS in South Korea to be conducted using the HIRA database, which represents the entire national population. This study shows that between the study period of 2010 and 2015, annual incidence remained similar while the number of prevalence increased 7.7% every year. Prevalence for men was 3.6 times higher than in women, and both women and men in their 30 s showed the highest prevalence. Men also showed 2.1 times higher incidence than women, and while the incidence was highest for men in their 20 s, it was so for women between ages 70 and 89. Men between ages 20 and 29 and women between ages 40 and 49 had the most diagnosis. With respect to income status, the highest prevalence and incidence of AS were found in the lowest income status.

To date, six hospital-based studies with large sample sizes (more than 100,000 population) have been conducted; five of these were confined to European countries and the other one was conducted in Asia^7–12^. Norway showed the highest age-adjusted prevalence and incidence (262.6 per 100,000 persons and 7.3 per 100,000 person-years, respectively) among the five European studies^8^. Greece had the lowest age-adjusted prevalence and incidence (29.5 per 100,000 persons and 1.5 per 100,000 person-years, respectively)^7^. The pathogenesis of AS remains unclear. While susceptibility to AS has been associated with the HLA-B27 gene, prevalence can differ due to differences in genetics, ethnic, and environmental factors^1,16,17^. Moreover, study bias due to the specific criteria for AS diagnosis or the particular study design can also affect calculated rates. The only study in Asia with a large sample size was conducted in Japan and reported a prevalence of 6.5 per 100,000 persons, the lowest prevalence reported globally^12^. However, the criteria for diagnosis were not standardized, and the response rate for survey participation at the referral institution was only 57.5%, indicating that the studied patient population might not have reflected the overall population. With respect to the study’s limitations, some regions had prevalence levels that differed by as much as six-fold.

In this study, the prevalence of AS in 2015 was 52.30 (95% CI, 51.68 to 52.92) per 100,000 persons. The prevalence increased linearly from 2010 to 2015 at a rate of 7.7% per year. A previous study in Norway reported that AS was 2.1 times more prevalent in 1990 than it had been in 1980^8^. This increase in prevalence might be affected by factors such as nationwide population increases, population age distribution, and mortality rates. Furthermore, since the time of AS diagnosis and treatment strategies can also affect prevalence, detecting prevalence trends of this disease is crucial for optimal national health care planning.

We found that AS was most common among patients in their 20 s in this study. This trend was also observed in men, in whom incidence peaked between the ages of 20 and 29; however, incidence for women was high between ages 20 and 29 with a gradual fall until age 50 then it turned around and reached peak among ages 70 and 89. One of the reasons may be that women receive diagnosis later than men. While diagnosis peaked for men in their 20 s, for women, diagnosis peaked in their 40 s. In particular, despite the fact that there is a population decrease with aging, diagnosis was very common for women in their 40 s and older. Many studies have reported that diagnosis of AS in women is delayed compared to men^18–20^. This delayed diagnosis in women is likely explained by the fact that AS is usually considered a predominantly male disease, and that AS manifests differently in women than in men^21^. Unlike men, women show symptoms of peripheral arthritis, implying that women with AS might have been misdiagnosed with seronegative rheumatoid arthritis in the beginning, then as the patients age receive a new diagnosis with AS^22^. In addition, several studies have reported radiographic differences of AS between men and women. Women tend to have less radiological spinal damage due to AS than men^22–25^. Elderly women, in particular, oftentimes do not receive accurate diagnosis for follow-up observation when they were younger because lower back pain does not receive much medical attention as it is considered a common pain in females. This demographic receives AS diagnosis after it has developed a radiological progression at an elderly age. A second reason may be the requirement of radiological change. Since AS has been included in the RID, detecting sacroiliitis is required for radiological diagnosis of AS. For some patients that have shown radiological changes in sacroiliitis with aging may have been diagnosed at an older age. Many studies have reported that diagnosis is delayed due to the radiological sacroiliitis criterion in the modified New York criteria^26,27^. Lastly, a bias in the study design may exist because the study uses the wash-out period of claims record after 2002. For instance, there may be medical claims related to AS during our study period for patients that may have been diagnosed before 2002 but had no treatment after 2002, and received a diagnosis again in 2015. In other words, bias may exist for older patients with a long pause claiming for insurance for AS, which makes it seems like it was their first diagnosis. However, it is hard to believe that no medical costs were claimed for 13 years. For AS patients diagnosed before 2002, patients only pay 10% of the medical costs using the AS diagnosis code even for prescription pain relievers for back pain; so having no medical cost for 13 years whatsoever and claiming for the first time in 13 years seems to be too rare of a case.

After rheumatoid arthritis, AS is the most common inflammatory rheumatic disease. However, few studies have quantitated the burden of the illness in relation to AS. Although symptom severity in AS varies among individual patients, frequently reported symptoms of AS include pain, stiffness, and fatigue^28^. These symptoms can be related to work disability. In a prospective study in Finland with 8 years of follow-up of 20 patients with AS, 10 to 15% of the patients reported having a work-related disability^29^. In a study from the Netherlands, the 658 patients with AS showed 11% lower labor force participation than the normal population and a 15% higher work disability rate^30^. Barlow *et al*. suggested that work disability should not be confined to employment alone, but rather should include a multidimensional notion encompassing work type, hours (from part-time to full-time), loss of promotional opportunities, use of sick leave, frequent job shifts, early retirement, and so on^31^. While this overall concept of work disability has been hypothesized to be highly associated with income status, this is the first study to report the prevalence and incidence of AS according to income status. We found that AS was three times more prevalent in medical aid recipients than in individuals with other income levels, and that the incidence of AS was five times higher in medical aid recipients. Other income statuses showed similar levels of prevalence and incidence. Medical aid recipients are individuals whose income is below the minimum cost of living. Therefore, the fact that medical aid recipients comprise 14.3% of all patients newly diagnosed with AS shows the substantial burden of this illness.

The strength of this study is that we used a nationwide database, which includes almost all of the South Korean population. Furthermore, after AS was included in the RID registration program in 2009, patients were able to receive more help with their medical costs. While this inclusion increased the interest in AS diagnosis, HIRA began reconfirming the diagnosis criteria of RIDs for reimbursement, meaning that these data are highly reliable. Nevertheless, the insurance claims data used in this study are associated with methodological limitations. If many AS patients were not treated at health care institutions, evaluations using insurance claim records can be underestimated. In South Korea, however, the NHI system is guaranteed for all of the South Korean population, allowing them easy access to clinics and hospitals. In fact, all patients with any symptom of AS would be receiving health service. Another limitation lies in the miscoding of ICD-10 diagnostic codes of the data in the insurance claim. Another study confirmed that diagnoses based on the RID database are more valid than other insurance data^32^. In addition, since we had to use administrative data, clinical status and symptom severity could not be studied here.

In conclusion, our results are reliable and can be generalized to a broader population. Moreover, they are based on the best currently available information on the epidemiology of AS in the South Korean population. The increasing trend of AS prevalence and the observation that 14.3% of all patients newly diagnosed with AS are medical aid recipients have significant implications for healthcare planning.
